# Youth Assets, Neighborhood Factors, Parental Income, and Tobacco Use: A Longitudinal Study of Health Disparities

**DOI:** 10.3390/ijerph191912330

**Published:** 2022-09-28

**Authors:** Eleni L. Tolma, Sara K. Vesely, Lindsay Boeckman, Roy F. Oman, Cheryl B. Aspy

**Affiliations:** 1 Department of Education Sciences, European University Cyprus, 6 Diogenous Street, P.O. Box 22006, Nicosia 1516, Cyprus; 2 Department of Biostatistics and Epidemiology, Hudson College of Public Health, University of Oklahoma Health Sciences Center, Oklahoma City, OK 73104, USA; 3 School of Public Health, University of Nevada, Reno, NV 89557, USA; 4 Department of Family and Preventive Medicine, University of Oklahoma Health Sciences Center, Oklahoma City, OK 73104, USA

**Keywords:** youth assets, tobacco use, health disparities, positive youth development

## Abstract

This study aimed to assess how the relationship between youth assets and future no-tobacco use among youth might differ according to race/ethnicity, neighborhood factors and socio-economic status. Five waves of annual data were collected from 1111 youth/parent pairs living in Oklahoma, USA who were randomly selected to participate in the Youth Asset Study (YAS). A marginal logistic regression model using all five waves of no-tobacco use, demographics, and their interaction was used to compare the change in tobacco use over time. Among 1111 youth, (Mean age = 14.3; 53% female; 39% White, 28% Hispanic, 24% Black, and 9% other), the percentage of youth tobacco use increased significantly from baseline to wave 5 (4 years after baseline) for all racial/ethnic groups and all parental income groups. Assets were prospectively associated with no tobacco use in the past 30 days for Black, White and Hispanic youth and for youth in all income categories (adjusted odds ratio range = 1.9–2.7). There was one statistically significant association between the neighborhood environment and future no tobacco use. To conclude, the protective effects of youth assets in terms of prevention of tobacco use among youth do not differ by youth race/ethnicity or parental income in the presence of neighborhood environmental factors.

## 1. Introduction

The USA has been successful in reducing cigarette smoking among youths during the last 30 years from 27.9% in 1991 to 6% in 2019 [1]. Successful strategies include the regulation by the U.S. Food and Drug Administration (FDA) in 2019 that increased the minimum age of sales for tobacco products from 18 to 21 years nationwide, the increased price of tobacco products, restricting access for youth to flavored tobacco products, the implementation of comprehensive smoke-free policies, and national anti-tobacco public health education media campaigns [2,3,4]. The term “tobacco products” relates to all products made or derived from tobacco including electronic cigarettes that are intended for human consumption as defined by the FDA [5]. However, it is also important to note that according to the World Health Organization (WHO), electronic cigarettes (e-cigarettes) are not considered tobacco products since they do not contain tobacco [6].

Despite these efforts an estimated 34 million American adults still smoke cigarettes [7]. What is more alarming, is that it is estimated that 6 million current American adolescent smokers will die prematurely due to tobacco use [8]. The rapidly developing adolescent brain is uniquely susceptible to nicotine addiction [9] and coupled with the fact that 90% of current adult smokers initiate smoking before the age of 19 years old [3], underscore the importance of youth-focused prevention efforts. In fact, in 2021, almost 1 in 10 (9.3%) (2.55 million) US students had used tobacco in the past 30 days, including 13.4% (2.06 million) for high-school students and 4.0% (470,000) for middle school students [10]. According to the same latest report [10] e-cigarettes were the most common tobacco product currently (i.e., past 30-day) used (7.6%) (2.06 million), followed by cigarettes (1.5%). In fact, e-cigarettes have been the most commonly tobacco product used among US youth since 2014 [10,11]. Even though at the time of the study e-cigarettes were not introduced, it is important to be mentioned because research has shown that the use of tobacco in any form (whether the product is combustible, smokeless or electronic) is unsafe [4].

The success of smoking prevention programming has not been equal among people of all racial/ethnic groups or income levels in the US. The Healthy People 2020 goal of reducing smoking prevalence to less than 12% has been achieved or exceeded only for some racial/ethnic groups (i.e., Asian, and some Hispanic/Latino with the exception of Puerto Ricans [12]), and for those with higher education (i.e., a 4-year college degree), and socio-economic status (i.e., income 400–599% higher of the poverty level) [13]. The progress was slower among populations with lower SES, being unemployed or blue-collar service workers [14,15].

In order to reduce health disparities, the root causes must be addressed. According to a report from the Office of Surgeon General, the determinants of tobacco use are organized into three ecological levels: biological, psychosocial and social/physical environmental factors [3]. In this study we will focus on two levels, the social/ physical environment factors and particularly the neighborhood environment, and the psychosocial factors through the concept of youth assets. Another model that has been used extensively in explaining tobacco use is the Biobehavioral model [16,17,18]. According to this model tobacco use is the product of interactions between biological (e.g., genetic vulnerabilities, health status) and behavioral (e.g., dependence, cue reactivity/craving) factors. This model has been used mostly in designing smoking cessation programs [19] rather than in designing preventative public health interventions.

Most recent studies that examined the roles of the neighborhood environment, family Socio-Economic Status (SES), and tobacco use among youth produced mixed results. For example, a study among African American youth found that objective neighborhood disorder and perceptions of neighborhood social cohesion were not significantly associated with past month tobacco use [20]. Another study by Mathur and colleagues [21] found that adolescents from families of lower SES were more likely to smoke over time if they resided in more affluent neighborhoods rather than in less affluent neighborhoods. On the contrary, another study found that living in less affluent neighborhoods was associated with early adolescent smoking; however, the presence of higher family income was uniquely protective against smoking [22]. To conclude, it is not clear how contextual factors, such as the neighborhood environment and family income impact tobacco use among youth. Therefore, this study will attempt to shed some more light on this area.

As mentioned earlier we will also focus on the psychosocial factors related to tobacco use and specifically, on the concept of youth assets. Youth assets are positive factors that reside within an individual such as competence and coping skills, as well as external to the individual such as parental support, institutional/organizational and community experiences [23]. Youth assets can help adolescents overcome the negative outcomes associated with poverty, and to become resilient [24]. The Youth Developmental Approach has been adopted by an increasing number of health-promoting initiatives [25,26] and posits that the possession of assets may simultaneously protect youth from engaging in a variety of risky behaviors.

Previous research established that youth assets are associated with decreased risk of tobacco use in both cross-sectional [27] as well as longitudinal studies [28,29]. In the first study [27] Non-Parental Adult Role Models and Use of Time assets were associated with no tobacco use for youth in one- and two-parent households. A second study [28] found that young adults who possessed certain assets such as Family Communication, Relationships with Mothers, Aspiration for the Future, Parental Monitoring or Responsible Choices had significantly higher odds of not using tobacco over time. In another study [29] Parental Monitoring and Positive Peer Role Models were the most important assets prospectively associated with no tobacco use for both genders. Finally, in another cross-sectional study [30] among middle and high-school students it was found that internal assets (e.g., positive identity, social competency) mitigated the negative effects of psychosocial risk factors of influence for tobacco use. In fact, internal assets were inversely associated with a 20% reduction in tobacco use [30].

This study is part of a group of studies that examined the relationship of youth assets, neighborhood conditions and family SES status longitudinally for several risky behaviors including alcohol use and binge drinking. A study by Oman et al. [31] showed that assets were prospectively associated with the absence of alcohol in the past 30 days and binge drinking in the past 6 months across the main three racial/ethnic groups (Non-Hispanic Black, Non-Hispanic White, Hispanic), and in most income levels. The study concluded that the protective effects of youth assets and the neighborhood environmental factors from youth alcohol use did not differ by youth race/ethnicity or parental income. Therefore, promoting youth assets in a positive neighborhood environment could be a mechanism to close the gap in health disparities related to alcohol use and binge drinking among youth. This study will attempt to determine if similarly, having a high number of youth assets and living in a positive neighborhood environment will protect youth from using tobacco over time across racial/ethnic groups and income levels.

## 2. Methods

### 2.1. Sampling and Data Collection

Census tracts in Oklahoma City, OK, USA and the immediate surrounding area (Oklahoma County) were stratified by income and race/ethnicity using 2000 census data and then randomly selected with the goal of obtaining a diverse community-based study population to follow through time. Twenty census tracts were included in the study. Door-to-door canvassing within the selected census tracts was conducted to obtain the baseline sample of youth and parents. One youth (age 12 to 17) and one parent from each consenting household participated in the study [32,33]. Each parent and child received $20 and $40, respectively, for the completion of each interview, and a $10 one-time payment for agreeing to be in the study, as a token of appreciation for their contribution to the study.

Data were collected from youth/parent pairs using Computer-Assisted Personal/Self-Interviewing procedures conducted in their homes by two-person interviewing teams. Youth completed the risk behavior items in private using the computers with wav sound files and headphones if necessary to minimize any potential reading problems. The youth interview was only available in English, however, the parent interview was available in both English and Spanish. Only 145 parents completed the interview in wave 1 in Spanish. Five waves of data were collected annually from the participants beginning with the baseline survey conducted in 2003/2004 and concluding in 2007/2008. A total of 1,111 youth/parent pairs participated in the study with a response rate of 61% [32,33]. The wave 5 response rate was 93% (1036/1111). The response rate across all five waves (i.e., valid youth interview at each of the five waves) was 89% (986/1111).

### 2.2. Measures

No-tobacco use in the past 30 days was the dependent variable (included in the analyses for waves 2 to 5). The independent variables were youth assets and the neighborhood environment (included in the analyses for Waves 1 to 4). Prospective associations between the independent and dependent variables were considered within the context of youth race/ethnicity and income-measured as the household’s Percent Federal Poverty Level (PFPL)-while controlling for other co-variates (youth sex and age, family structure, household income, parental education, crowded household, and household wealth).

Demographic data, including youth age, sex, race/ethnicity, and family structure as well as information on asset data and tobacco use were collected from the youth. At baseline, youth race/ethnicity was coded non-Hispanic White, Hispanic, non-Hispanic Black and other. In the race/ethnicity specific and overall analyses, youth who were identified as Native American, Asian and other races (*n* = 110 excluded) were excluded from the sample due to their relatively few numbers. Family structure was assessed at baseline with response options of one- or two-parent household; at subsequent waves, the youth could respond “independent” if they had lived alone for at least 6 months.

Seventeen youth assets were assessed via multi-item constructs with established validity and reliability. Seven assets operated at the individual level, four at the family level, and six at the community level. The asset constructs were conceived and developed based on literature reviews, previous research, and on psychometric testing [33]. Items representing each asset were summed and divided by the number of items to create a score ranging from 1 (almost never/strongly disagree/very low participate in positive event or behavior) to 4 (almost always/strongly agree/very high). A youth was determined to have the asset of his/her mean score was 3 or higher. The median number of assets possessed by youth in the study was 12. Therefore, based on the median number of assets, a high (12 or more assets) versus a low (11 or less assets) asset variable was created. The reliability of the asset constructs was adequate (Cronbach’s alphas > 0.70 for 11 assets, >0.60 and ≤0.70 for four assets, and ≥0.55 and ≤0.60 for two assets).

Tobacco use was assessed by the question “During the past 30 days have you used any tobacco (smoked, dipped, or chewed)?” which is a standard item used in the Youth Risk Behavior Survey [34]. Possible responses were “Yes” or “No”.

Demographic data were collected from the parents including parental education, parent income, crowded household, household wealth, household income and the number of people living on the household income. Household income and the number of people living on the total household income were used to calculate PFPL. The neighborhood environment data was primarily collected from the parents; however, objective trained raters also assessed the neighborhood environment.

Parental education was stratified into 3 categories: both parents had less than a high-school education; at least one parent had completed high school, GED or some college; and at least one parent had a bachelor’s degree or higher. Yearly household income was also stratified into 3 categories: less than $35,000, $35,000 to $62,000 and greater than $62,000.

Household wealth was determined using two methods. First, by reporting the number of assets a family owned including a home, savings, tax deferred plans (e.g., 401K), real estate investments, etc. There were 7 different kinds of assets and each of these 7 categories contributed a value of 1 to the household wealth score if the asset was owned. The second method used to calculate household wealth was by asking the parent to respond to the question “Suppose you were to sell all your major processions (including your home), turn all your assets into cash and pay all your debts, would you have something left over, break even or be in debt?” The response to this item resulted in a value of 1 (“left over”), 0 “break even”), or −1 “be in debt” to the sum of the household wealth average (−1 to 8). The PFPL variable, which served as the primary indicator of household income, was assessed by dividing total household income by the number of people supported by total household income. Four poverty levels were created: 0–100%, 101–200%, 201–300% and >301%.

The crowded household variable was created by calculating the number of rooms in the home and the number of people living in the home. Households with greater than one person per room were coded as a crowded household.

Neighborhood context was assessed annually by trained raters who conducted windshield tours of each census tract included in the study. The objective assessment of the neighborhood was assessed via the Broken Windows (BW) survey which was adapted from previous research [35]. The survey assesses neighborhoods according to the condition of the dwellings, and the amount of trash, graffiti, and abandoned cars. The BW survey score ranged from 0 (neighborhood in poorer condition) to 12 (neighborhood in better condition). The Spearman correlation coefficient for the test–retest reliability of the BW survey was 0.83 and the intraclass correlation was 0.80.

Neighborhood crime and safety was assessed with five items such as, “There is crime and violence in your neighborhood.” The Cronbach’s alpha was 0.87. Neighborhood services were assessed with four items such as, “There is poor police protection in your neighborhood.” The Cronbach’s alpha was 0.69. Possible responses for the neighborhood concern questions ranged from one (strongly agree) to four (strongly disagree). Some of the neighborhood concerns items were adapted from previous research and some were created by the research team [36]. Both neighborhood concern variables were analyzed as dichotomous variables: 1 to <3 (no) versus 3–4 (yes).

Neighborhood support was assessed with five items such as, “About how often do you and people in your neighborhood watch over each other’s property?” [37]. Responses ranged from one (almost never) to four (almost always). The Cronbach’s alpha was 0.77. Neighborhood support was analyzed as a dichotomous variable:1 to <3 (low) versus 3–4 (high). Sense of community was assessed using the Psychological Sense of Community (PSOC) scale [38]. The PSOC scale included seven items such as “People in this neighborhood get along with each other.” Possible responses ranged from one (strongly disagree) to four (strongly agree). Cronbach’s alpha for the PSOC scale was 0.84. Sense of community was analyzed as a dichotomous variable: 1 to <3 (low) versus 3–4 (high). Finally, informal social control was assessed with five items such as, “How likely is it that your neighbors will become involved if children are skipping school and hanging out on the street corner?” [39]. Responses for the scale ranged from one (very unlikely) to four (very likely). The Cronbach’s alpha was 0.82. Informal social control was analyzed as a dichotomous variable: 1 to <3 (low) versus 3 to 4 (high).

### 2.3. Statistics

Descriptive analysis was done among demographics variables at baseline. To assess the trajectory of tobacco use by race/ethnic group and level of PFPL a marginal (“population average”) longitudinal regression model was conducted. As a second step, in the above marginal regression model, we added youth assets in their dichotomous form while evaluating and controlling for the nine youth and parental demographics with family structure, parental income and parental education as time-varying and lagged.

Next, in order to examine the prospective association between youth assets and no tobacco use in the presence of environmental factors, we conducted prospective longitudinal analyses using marginal logistic regression analysis (Generalized Estimating Equations method) between assets (waves 1–4) and environmental factors (waves 1–4) with no tobacco use in the following wave (waves 2–5) while controlling for demographics. Assets and environmental factors were analyzed as time-varying and lagged and included in all models. A diagonal working covariance matrix was used as recommended by Pepe and Anderson when covariates vary over time [40]. We analyzed 2-way interactions between race/ethnicity and wave, assets, and environmental factors, as well as 2-way interactions between federal poverty level and wave, assets, and environmental factors. No significant interactions were detected. Finally, to evaluate health disparities we, a priori, chose to perform stratified analyses by race/ethnicity and by percent federal poverty level. The analysis was performed using SAS 9.3. An alpha of 0.05 was used except for interaction evaluation, for which cases an alpha of 0.005 was used to control type I error.

## 3. Results

### 3.1. Data

At baseline 1,111 youth were interviewed; 1001 youth who reported their race as non-Hispanic White, non-Hispanic Black, or Hispanic were included in our analysis. Our analysis lagged the demographics, assets, and environmental variables at wave 1 with the tobacco outcome at wave 2 and then wave 2 with wave 3, etc., resulting in four time points for data analysis. There were no missing data for youth age, gender, or race/ethnicity or family structure. There were no missing values for parental education at baseline; however, in subsequent waves when parental education was missing, the response from the prior wave was carried forward.

### 3.2. Demographics

Fifty-three percent of the participants (*n* = 1001) included in the analysis were female. The sample’s race/ethnicity was 43% Non-Hispanic White, 26% Non-Hispanic Black, and 31% Hispanic. Household income was 50% with income less than $35,000, 30% $35,000 to $62,000 and 20% >62,000; 69% of the youth were living with both parents and 31% were living with one parent. Almost two thirds of the youth (58%) possessed 12 or more assets at wave 1. Descriptive data are shown in Table 1.

### 3.3. Trends in Tobacco Use by Race/Ethnicity and by Federal Property Level (PFPL)

**Figure 1:**
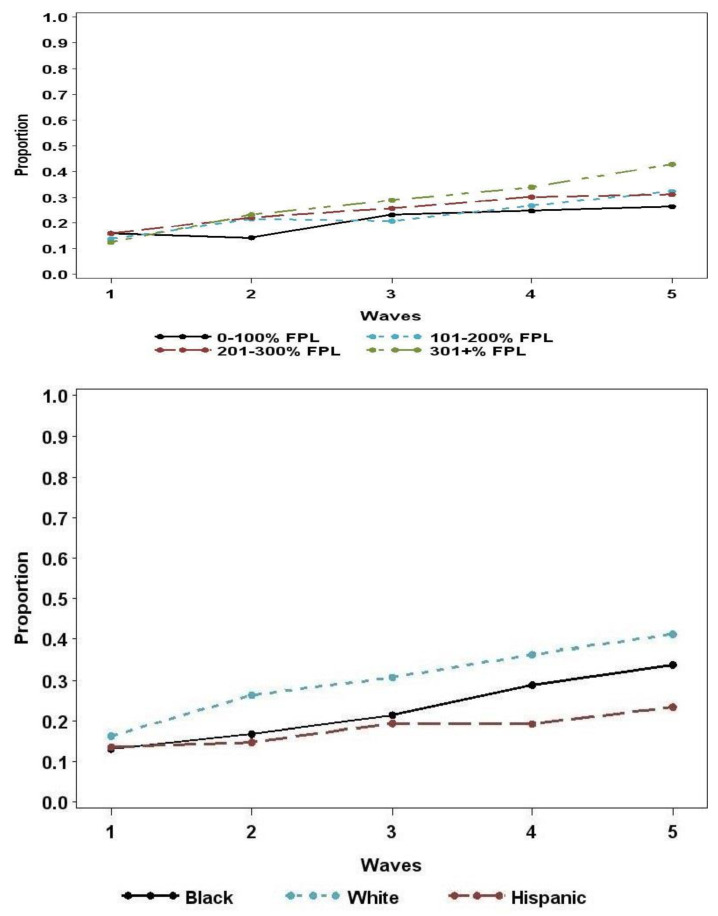
Trends in Youth Tobacco Use by Percent Federal Poverty Level and Race/Ethnicity.

The proportion of youth who used tobacco increased over the five waves of the study (Figure 1). Specifically, in wave 1 the proportion was 14%, in wave 2, 21%, in wave 3, 25%, in wave 4, 29% and in wave 5, 34%. Overall, there was a statistically significant association between time/wave and tobacco use (*p* < 0.0001). The odds of tobacco use significantly increased for each subsequent wave compared to wave 2.

Tobacco use ranged from a low of 13% for Non-Hispanic Black youth at Wave 1 to a high of 41% for Non-Hispanic White youth at Wave 5. In addition, tobacco use ranged from a low of 12% for youth in the >301% PFPL category to a high of 43% of youth in the same category income (Figure 1).

### 3.4. Asset, No Tobacco Use, and Race/Ethnicity

We assessed the impact of the number of assets (high vs. low), wave and demographics in the prediction of no tobacco use through a multivariate analysis (Table 2). The results showed that a high number (i.e., 12 assets and above) of assets was significantly associated with no tobacco use (*p* < 0.001) (OR = 2.2, 95% confidence interval (CI): 1.8, 2.7).

Race/ethnicity was significantly associated with no tobacco use (*p* < 0.0001) with the odds of not using tobacco 1.9 times greater in Non-Hispanic Black youth as compared to the Non-Hispanic White youth, and the odds of not using tobacco 1.8 times greater for Hispanic youth as compared to the Non-Hispanic White youth.

### 3.5. Asset, Tobacco Use and Income Levels

None of the income levels as measured by the PFPL variable were associated with no tobacco use in all three levels. More information about the relationship between assets, no tobacco use and other demographics can be found in Table 2.

### 3.6. Asset, Tobacco Use by Race/Ethnicity in the Presence of Neighborhood Conditions

Non-Hispanic White, Non-Hispanic Black, Hispanic and all youth were significantly less likely (*p* < 0.05) to use tobacco if they had 12 more assets (Table 3) even after adjusting for demographic and neighborhood conditions. The adjusted odds ratios (AORs) ranged from 2.0 (CI: 1.4, 2.7) for Non-Hispanic White youth to 2.5 (CI: 1.6, 3.7) for African American youth. Neighborhood conditions were not related to tobacco use for youth for any race/ethnicity and for all youth.

### 3.7. Asset, Tobacco Use by Percent Federal Poverty Level in the Presence of Neighborhood Conditions

At all income levels, youth with 12 or more assets were significantly less likely (*p* < 0.05) to use tobacco (Table 4). The AORs ranged from 1.9 (CI: 1.3, 2.8) in the lowest income level to 2.7 (CI: 1.8, 3.9) in the highest income level. There was only one significant association related to neighborhood conditions. Youth whose parents were in the highest income group and had a stronger sense of community had a 50% reduction in the odds not to use tobacco compared to the youth whose parents were in the highest income group and had a weaker sense of community (OR: 0.5, CI: 0.3, 0.8).

## 4. Discussion

This study had dual purposes. First, to assess the role that youth assets play in the prediction of tobacco use within a diverse random group of urban youth in Oklahoma while taking in consideration certain demographic variables including race/ethnicity, family structure, parental education, and family income. Second, to assess the role youth assets play in the prediction of tobacco use through the stratification of both family income and race/ethnicity in the presence of neighborhood conditions as measured by both objective (i.e., broken windows survey) as well as subjective measures (e.g., neighborhood concerns) via the parental interviews. The information derived from this study can help professionals who work in tobacco use prevention by focusing on the underlying social-environmental disparities that produce tobacco-related health disparities among youth.

From a behavioral perspective our results do not reflect the national picture which was not a surprise. Tobacco use ranged from 14% in wave 1 to 34% in wave 5 among youth of ages 12–17 at baseline. The current rate based on the most recent National Youth Tobacco Survey [10] is 9.3% for all students, and 13.4 % among high-school students. Considering that by wave 5, most of the youth in our study were already in high school, the proportions are different, with the youth in our study having a higher proportion (34% vs. 13.4%). This discrepancy can be attributed to the differences in the year of the data collection between the two studies, as well as the design of the studies (community-based vs. school-based).

Another interesting observation is that white youth across time are more likely to use tobacco (39%) compared to youth from other ethnic groups as supported by the literature. According to the National Youth Tobacco Survey in 2021 white youth are more frequent tobacco users (11%) compared to Hispanic (7.4%) and Black (8.2%) youth [10]. Previous studies also supported that Black adolescent are less likely to use alcohol and tobacco than their white counterparts [41,42].

Another interesting finding is that wealthier youth are more likely to use tobacco over time than youth from other income categories despite studies showing the opposite result [22,43]. This discrepancy could be due to methodological differences. Specifically, the first study [22] took place in a school setting with an over-presentation of high crime neighborhoods whereas the second study [43] took place in France, where the socio-cultural context might be different than the one in this study. However, in the presence of a high number of youth assets, family income was not influential in the prediction of tobacco use over time. The results suggest that the presence of youth assets might mitigate the effect of income in the prediction of tobacco use among youth.

The results of this study clearly give support to the existing literature [28,29,30] that states that youth assets have a protective effect from tobacco use among youth over time. However, one of the questions that arise from the current literature is “does this protective effect of youth assets remain strong across racial/ethnic groups, income levels, and neighborhood conditions (positive or negative)”? Furthermore, if yes, who among the racial/ethnic groups or family income groups might benefit the most?

The results of this study have shown that youth assets retain their long-term protective effect regardless of the neighborhood conditions the youth live in and across ethnic/racial groups, with African American youth as the group that benefits the most. Neighborhoods through their networks and infrastructure can be a source of pride, a source of positive resources and a source of positive role modeling. On the other hand, poor neighborhoods can be a source of violence, fear, and powerlessness that generate chronic stress and despair for the youth and thus push them to coping mechanisms such as alcohol and tobacco use. As discussed earlier there are mixed results of the role that the neighborhood environment plays in tobacco use on adolescent smoking [20,21,22]. In this study we have shown that a high number of youth assets can protect youth from the adverse neighborhood conditions they might be experiencing, and most importantly that youth assets retain their long-term protective effect regardless of the neighborhood conditions the youth live in.

Similarly, youth assets seem to be protective of tobacco use across family income categories with youth from the wealthiest families benefiting the most. Coupled with the fact that over time youth from higher income families were more likely to use tobacco than youth from other income groups, this suggests that youth assets can benefit not only youth from low-income families as one might expect, but also those youth who are already wealthy (and have many tangible resources). Therefore, an emphasis on promoting youth assets could be beneficial for all youth regardless of their socio-economic background.

One unexpected finding was that youth who came from wealthy families and lived in neighborhoods with a greater sense of community were more likely to use tobacco. Parents who indicated that they experienced greater sense of community were also more likely to indicate that they shared similar values with their neighbors and cared about how they were perceived by their neighbors. It is possible, even though we did not assess the family smoking environment, that this group of parents also smoked, through the peer effect exerted by their social networks [44], and therefore they created a more permissive family environment in relation to tobacco use for their children. Further qualitative research can shed more light in this finding.

From a programmatic perspective the results of this study show that the neighborhood context the youth live in is important; however, what is more important is developing programs within the neighborhoods with a focus on youth assets. Based on an earlier study, two assets that seemed to be important regarding tobacco use among boys and girls were Parental Monitoring and Positive Peer Role Models [29]. Earlier studies have shown that a combination of social competence and social influences approaches as a component of a school-based “resilience” intervention were effective in reducing tobacco use [45,46]. However, a more recent systematic review has found that school-based “resilience” interventions targeting tobacco use did not have an overall intervention effect [47]. Therefore, the results of the study show that focusing on the promotion of youth assets across racial/ethnic groups and families of different SES could be an alternative approach that includes the whole community and not just the school setting.

The study had a few limitations. One study limitation was that socially acceptable answers may have been given in response to some questions. However, since youth were allowed to self-administer the risk-behavior items, the number of socially acceptable responses may be minimal. A second limitation was the study’s 61% response rate. The study was atypical in that participants were recruited via door-to-door canvassing of every household in the randomly selected census tracts with the goal of obtaining a community-based sample that was racially/ethnically and economically diverse. The representativeness of the study sample was evaluated by comparing by census tract the participants’ race/ethnicity and total family income data (collected in 2003/2004) to 2000 census data for race/ethnicity and median family income. Family income was not different from the study sample in 17 of 20 census tracts; in the other 3 the family income census data was lower. Youth participant race/ethnicity was compared to the overall race/ethnicity of the census tract; it was not different in 5 tracks and in the other 15 the percent of NonWhites, in particular Hispanics, was generally higher in the study sample compared to the census data. These race/ethnicity differences could be explained by the fact we intentionally oversampled minority households as well as due to the issue of an indirect comparison (race/ethnicity of the youth study participants compared to race/ethnicity of the total population of the census tract). Another methodological limitation is the fact that during the analysis we did not account for any possible data clustering since we sampled by census tracts and census tracks do not correspond to any “neighborhoods”. A final limitation stems from the fact that this study took place in an urban setting. Recent research [48,49] has shown that living in rural areas can be an independent risk factor for tobacco use among youth. Therefore, future similar studies should take place in a rural setting to investigate if indeed the relationship between youth assets, demographics and neighborhood environment are the same as found in this study.

## 5. Conclusions

Τhe protective effects of youth assets and neighborhood environmental factors in terms of prevention of tobacco use among youth do not differ by youth race/ethnicity or parental income. African American youth and youth from families with the highest income level seem to benefit the most from the presence of a high number of youth assets in their lives.

## Figures and Tables

**Table 1:** Baseline demographic characteristics of the total sample, (*n* = 1111).

		Total Sample (*n* = 1111)
Demographic	Response	*n* (%)
Age in years (mean, sd)		14.4 (1.6)
Race/Ethnicity	Non-Hispanic African American	261 (24%)
	Non-Hispanic White	434 (39%)
	Hispanic	306 (28%)
	Non-Hispanic Other	104 (9%)
Parent Education	both < HS *	178 (16%)
	one HS/no college	624 (56%)
	at least 1 college	309(28%)
Family Structure	Two Parent	773 (70%)
	One Parent	338 (30%)
Household Income	<$35,000	543 (49%)
	$35,000–$62,000	330(30%)
	>$62,000	225 (21%)

* HS = High-School.

**Table 2:** Effect (odds ratios) of all covariates on odds of youth not using tobacco, multivariate model.

Covariates	OR (95% CI)	*p*-Value
Wave 5 vs. Wave 2	**0.5** (0.4, 0.6)	<**0.0001**
Wave 4 vs. Wave 2	**0.6** (0.5, 0.7)	
Wave 3 vs. Wave 2	**0.8** (0.7, 0.9)	
101–200% FPL vs. 301+% FPL	1.3 (0.8, 2.0)	0.4521
201–300% FPL vs. 301+% FPL	1.3 (0.9, 2.0)	
0–100% FPL vs. 301+% FPL	1.4 (0.8, 2.4)	
Race-Non-Hispanic African American vs. Non-Hispanic White	**1.9** (1.4, 2.5)	<**0.0001**
Race-Hispanic vs. Non-Hispanic White	**1.8** (1.3, 2.6)	
Male vs. Female	**0.6** (0.5, 0.8)	**0.0001**
One parent vs. Two parent household	0.9 (0.7, 1.2)	0.4648
Independent vs. Two parent household	0.8 (0.5, 1.2)	
Parent income-<$35,000 vs. >$62,000	0.9 (0.7, 1.3)	0.5941
Parent income-$35,000–$62,000 vs. >$62,000	0.8 (0.5, 1.2)	
Parent education-1+ with HS/GED/some college vs. 1+ with college degree	**0.7** (0.5, 0.9)	**0.0014**
Parent education-Both with less than HS vs. 1+ with college degree	1.0 (0. 7, 1.6)	
Crowded household	1.4 (0.9, 2.1)	0.0956
Sum of 8 wealth indicators	1.0 (0.9, 1.1)	0.981
14–15 years vs. 12–13 years at Wave 1	**0.7** (0.5, 0.9)	<**0.0001**
16–17 years vs. 12–13 years at Wave 1	**0.4** (0.3, 0.5)	
High number of assets	**2.2** (1.8, 2.7)	<**0.0001**

**Table 3:** Results of Logistic Regression Analyses of Assets and Neighborhood Factors on No Tobacco Use for All Youth and by Youth Race/Ethnicity.

	Adjusted Odds Ratio * (95% CIs)				
Youth Race/Ethnicity		All Youth	Non-Hispanic White	Non-Hispanic African American	Hispanic
Assets	**No Tobacco Use**	**2.2** (1.8, 2.7)	**2.0** (1.4, 2.7)	**2.5** (1.6, 3.7)	**2.4** (1.6, 3.4)
Broken Windows	**No Tobacco Use**	1.0 (0.9, 1.0)	1.0 (0.9, 1.1)	1.0 (0.9, 1.0)	0.9 (0.9, 1.0)
Neighborhood Support	**No Tobacco Use**	0.9 (0.7, 1.2)	1.0 (0.7, 1.4)	0.9 (0.5, 1.5)	1.1 (0.6, 2.2)
Informal Social Control	**No Tobacco Use**	1.0 (0.8, 1.2)	1.0 (0.7, 1.4)	0.7 (0.5, 1.2)	1.3 (0.8, 2.0)
Sense of Community	**No Tobacco Use**	0.8 (0.7, 1.0)	0.7 (0.5, 1.0)	0.7 (0.4, 1.1)	1.1 (0.8, 1.6)
Neighborhood Concerns Services	**No Tobacco Use**	0.9 (0.8, 1.1)	0.9 (0.6, 1.1)	0.9 (0.6, 1.3)	1.0 (0.7, 1.5)
Neighborhood Concerns Crime	**No Tobacco Use**	1.1 (0.9, 1.3)	1.0 (0.7, 1.3)	1.1 (0.7, 1.6)	1.3 (0.9, 1.9)

* Adjusted for study wave, youth sex age and race/ethnicity, family structure, parent income and education, crowded household, % poverty level, household wealth, neighborhood factors, and assets.

**Table 4:** Results of Logistic Regression Analyses of Assets and Neighborhood Factors on No Tobacco Use by Percent Federal Poverty Level.

	Adjusted Odds Ratio* (95% CIs)				
**Percent Federal Poverty Level**		**0–100%**	**101–200%**	**201–300%**	**301+%**
Assets	**No Tobacco Use**	**1.9** (1.2, 2.8)	**2.1** (1.4, 3.1)	**2.1** (1.4, 3.2)	**2.7** (1.8, 3.9)
Broken Windows	**No Tobacco Use**	0.9 (0.8, 1.0)	1.0 (0.9, 1.1)	1.0 (0.8, 1.1)	1.0 (0.9, 1.1)
Neighborhood Support	**No Tobacco Use**	1.2 (0.6, 2.6)	0.8 (0.5, 1.4)	1.2 (0.7, 2.3)	0.8 (0.6, 1.3)
Informal Social Control	**No Tobacco Use**	1.1 (0.7, 1.7)	0.9 (0.6, 1.3)	1.3 (0.9, 2.0)	0.7 (0.4, 1.1)
Sense of Community	**No Tobacco Use**	1.0 (0.7, 1.5)	0.7 (0.5, 1.1)	1.2 (0.8, 1.8)	**0.5** (0.3, 0.8)
Neighborhood Concern Services	**No Tobacco Use**	1.0 (0.7, 1.5)	0.7 (0.5, 1.1)	1.1 (0.8, 1.6)	0.9 (0.7, 1.3)
Neighborhood Concerns Crime	**No Tobacco Use**	1.2 (0.8, 1.8)	1.1 (0.8, 1.6)	1.3 (0.9, 2.0)	1.0 (0.7, 1.4)

* Adjusted for study wave, youth sex, age and race/ethnicity, family structure, parent income and education, crowded household, % poverty level, household wealth, neighborhood factors, and assets.

## Data Availability

The data that support the findings of this study are available from the authors upon reasonable request.
